# The General Use of Histamine Antagonists

**Published:** 1950-07

**Authors:** Robert P. Warin

**Affiliations:** Dermatologist, United Bristol Hospitals; Clinical Teacher in Dermatology, University of Bristol


### THE GENERAL USE OF HISTAMINE ANTAGONISTS

### *With special reference to Therapy in Dermatological Disorders*

BY

ROBERT P. WARIN, M.D., M.R.C.P.

*Dermatologist, United Bristol Hospitals;*

*Clinical Teacher in Dermatology, University of Bristol.*

---

The list of histamine antagonist drugs grows, and a certain amount of confusion and debate exists as to which drug is the most satisfactory and which disorders can be improved by their administration. Recently the equipotent doses of the commonly available preparations have been determined (Bain, Broadbent and Warin, 1949, and Bain, 1949). In this work weals have been induced in the skin of man by intradermal injections of histamine. The size of the weal is less if the injection is made at a suitable interval after one of the drugs has been taken by mouth. By using a series of subjects and carefully measuring the areas of weals provoked before and after giving different doses of the various agents it has been possible to make an accurate comparison of the effectiveness, weight for weight, of the drugs as histamine antagonists in the skin of man.

Table I gives the names of the agents which are at present available on the market in this country and the doses that will give the same degree of antihistaminic effect. These equipotent values refer to the administration of single doses only. It is interesting that the drugs in this series, although having the same clinical action and often the same side effects, have for the most part quite different chemical structures.

Apart from the degree of effect from single doses the agents vary considerably in their length of action (Bain, Hellier and Warin). The lengths of action on the skin of man following ingestion have been determined in a similar manner to that used in measuring the weight-for-weight potency. For the purpose of comparison it is convenient to take the period from ingestion to the time when only 50 per cent. of the anti-histaminic effect remains. Not enough work has yet been carried out to give the exact times for each drug, but the following figures can be considered as a practical guide (Bain, 1949): Benadryl, 8 to 12 hours; Antistin, 4½ to 6½ hours; Anthisan, 5 to 7 hours; Pyribenzamine, 4 to 6 hours; Phenergan, 24 to 36 hours. Histantin appears to have a slightly shorter action than Phenergan.

TABLE I

THE HISTAMINE ANTAGONIST DRUGS AVAILABLE ON THE MARKET IN THIS COUNTRY

| Name of drug | Manufacturer | Comments | Equivalent doses (mg.) |
|---|---|---|---|
| Benadryl .. | Parke Davis .. | — | 200 |
| Antistin .. | Ciba .. .. | — | 350 |
| Histostab .. | Boots .. .. | Essentially the same substance as Antistin. | 350 |
| Anthisan .. | May & Baker | Introduced in France as Neo-Antergan. | 150 |
| Pyribenzamine | Ciba .. .. | There is only a slight difference chemically between this substance and Anthisan. | 160 |
| Phenergan .. | May & Baker | — | 25 |
| Histantin .. | Burroughs Wellcome. | — | 50–100 |
| Di Paralene .. | Abbott .. | Essentially the same substance as Histantin. | 50–100 |
| Thephorin .. | Roche .. | — | Not known |

Some other drugs are in use abroad, notably in America. These seem to have no important advantages over the range available in this country and include Histadyl (Eli Lilly), Chlorothen (Lederle), Anthallan (Medico Chemical Corps) and Trimeton (Schering Corp.). A complete list of antihistamine preparation has recently been prepared (*Pharmaceutical Journal*, February 18th, 1950, p. 139).

It is of course obvious that when using a drug with such a long action as Phenergan repeated doses will build up a total effect much greater than the total effect from repeated doses of a shorter acting agent. It is therefore apparent that the relative potencies for single doses as given in Table I do not give an accurate comparison of the drugs when repeated doses are given. For example, compare Anthisan and Phenergan. When single doses were employed, 150 mg. of Anthisan were found to be equipotent with 25 mg. of Phenergan. But Phenergan has 3 to 4 times the duration of action of Anthisan, so when the drugs were compared in a clinical study, in which they were given daily for at least a week, it was found that 350 mg. of Anthisan per day was equipotent with 25 mg. of Phenergan (Bain, Broadbent and Warin, 1949).

As has been mentioned Phenergan has been shown to have lost only half of its effect approximately twenty-four hours after ingestion. This property has enabled the drug to be used in a single dose during a twenty-four-hour period, and if this dose is given at night any attendant drowsiness will be most marked throughout the hours in which the patient would normally sleep.

An important limiting factor in any drug of this series is the tendency to give rise to side effects. The main side effect with all the drugs so far studied is the sensation of drowsiness, lassitude or dullness. Dryness of the mouth is common, but is usually not a significant inconvenience. Nausea, vomiting and sometimes diarrhoea may be provoked by Anthisan or Pyribenzamine, but are less frequent if the drugs are not given on an empty stomach. Phenergan may give rise to a curious aching and weakness of the legs. Other symptoms from therapeutic doses of these drugs have been reported but occur infrequently. These include restlessness, dizziness, anorexia, headache, blurred vision and dysuria. Patients vary considerably in their reaction to the side effects of drowsiness and lassitude. One person may not be able to tolerate 300 mg. of Anthisan per day, whereas another may be able to take 1200 mg. of Anthisan per day without undue toxic symptoms. Sensitive intelligent patients who are ambulatory are more likely to be affected than the more phlegmatic, particularly if they are lying in bed. If increasing doses of any of the drugs are given then eventually all patients complain of these side effects. It has been noticed that many patients develop a tolerance to such side effects as drowsiness and lassitude, often after a few days. In this connection it is important to realize that tolerance to the specific antihistaminic effects apparently does not occur even after many months' continuous therapy (Warin, 1948).

The tendency for the different drugs to cause side effects varies considerably, and this variation is important in comparing their value for clinical use. It is obvious that a comparison of the incidence of side effects must be made with equipotent doses of the drugs. However, a number of misleading comparisons have been reported in which the doses employed have obviously had very different antihistaminic effects.

When equipotent doses are employed the incidence of side effects is greatest with Benadryl. Antistin shows a lower incidence than Benadryl, but greater than Anthisan and Phenergan when the latter is used in a single nightly dose.

It seems most likely that the new drug Histantin (Burroughs Wellcome & Co.) may in its turn have the lowest incidence of side effects. From a preliminary assessment of a clinical comparison, being undertaken at the Bristol Royal Hospital, Histantin appears to be superior to both Anthisan and Phenergan.

Two further points in the practical use of these drugs must be mentioned. Individual patients vary considerably in the degree of histamine antagonism given by the same dose of one of the drugs and also in length of time that the effect of the drug will remain (Bain, Hellier and Warin, 1948). Thus the same degree of histamine antagonism given in one patient by, for example, 300 mg. of Anthisan per day may not be given in another until a dose of perhaps 1200 mg. is reached. If a patient shows a small antihistaminic effect with one drug of the series a small effect will generally be given by the other drugs. In the practical administration of the drugs this variation in response is usually allowed for by giving the patient an average dose, and if the symptoms are not controlled the dose is raised until either the desired effect is brought about or side effects become too great.

In addition to this variation in individual response to the drugs the strength of stimuli evoking the treated disorder will vary from patient to patient and will fluctuate in the same patient. An example of this is given by a patient with chronic urticaria suppressed by 300 mg. of Anthisan per day who developed an outbreak of weals in spite of continuing to take the same dose of the drug. This attack was found to have coincided with a considerable emotional upset. The fresh outbreak of urticaria was suppressed by raising the dose of Anthisan to 600 mg. per day for two weeks, after which period it was found that the eruption could once more be suppressed by 300 mg. per day (Warin, 1949).

The series of antihistaminic drugs has now been in general use for two to three years. Very large quantities have been administered in all sorts of disorders. The indications for use are still to be defined and very little carefully controlled work has been carried out. Many difficulties are experienced in deciding whether a condition is affected by the drugs. In this connection it seems probable that in a number of disorders reported improvement by histamine antagonist therapy may have been entirely due to the sedative side action of the drugs.

With regard to disorders involving the skin the only conditions irrefutably improved by the histamine antagonists are those with urticarial weal formation. These include acute urticaria from various causes, chronic urticaria, dermographism, giant urticaria (angio-neurotic oedema) and papular urticaria. Probably if a large enough dose of the drugs can be given a response can be obtained in all cases. For example, in a series of forty cases of chronic urticaria, all were found to respond, although in one or two it was found necessary to give a dose which caused too great a degree of drowsiness to warrant prolonged therapy (Warin, 1949). In some cases of acute urticaria the causative stimuli provoke such a severe response that very large doses of the drugs are required. Comparatively large doses may also be required to control attacks of papular urticaria, probably because children as a rule are relatively resistant to the drugs, and the degree of antihistaminic effect from average doses may not be sufficient to control the eruption. With those disorders characterized by recurrent short attacks, such as giant and papular urticaria, there is some evidence that the attacks can be lessened if the drugs are given as soon as possible after the attack commences. This will, of course, greatly help in the practical therapy of these conditions, as otherwise administration of the drugs for very prolonged periods will be necessary in order to control attacks that might only occur every few weeks or months. With chronic urticaria the drugs essentially suppress the weals, which usually return after stopping the tablets when the concentration of the drug drops below a certain level. Therapy may have to be continued for prolonged periods, and some patients have taken the drugs continuously for a year or even two years. The hope in treatment is that during the time the weals are suppressed the tendency to urticaria will be reduced by other therapeutic measures or will die out spontaneously. In a series of thirty cases that had been followed up, the tendency to urticaria died out whilst on suppressive therapy in eleven and was considerably lessened in six; in thirteen the tendency to urticaria was unchanged and suppressive therapy continued (Warin, 1949). As would be expected, the tendency is more likely to persist in those patients who have had the urticaria for a long time prior to therapy.

In the eczema-dermatitis disorders the value of the antihistamine agents is debated, and there is at present no controlled work recorded, which will provide a clear decision as to the effect of the drugs on these conditions. It is suggested that improvement due to the specific effects of the drugs only occurs when it is apparent that the eruption is associated with dermal oedema, as is often seen in dermatitis due to contact with irritants to which the skin has developed an allergic sensitivity.

The assessment of the value of the drugs in pruritus from various causes has been difficult. Initially the reports suggested a specific anti-pruritic effect. The itching of scabies, jaundice, eczema, and many other conditions was said to be relieved. More recent reports seem less convincing, and in a controlled investigation which is being carried out in the Bristol Royal Hospital it seems that any anti-pruritic effect is probably due to the sedative side effects of the drugs.

It appears that the histamine antagonists have no specific effect on rosacea, dermatitis herpetiformis, lupus erythematosus, lichen planus or pemphigus. The position with regard to erythema multiforme and actinic prurigo is not quite clear, although any effect is probably slight. A few cases of post-herpetiform neuralgia are reported to have been relieved, but the results are inconstant. Some cases of periarteritis nodosa are reported to have been improved, but again the results seem to vary considerably.

### Summary

1. The histamine antagonists at present available in this country are compared.

2. Phenergan when given in a single nightly dose every twenty-four hours, Anthisan and Histantin all seem to give a low incidence of side effects in average clinical doses. Histantin may prove to be the most useful drug in this respect.

3. Employing single doses, Phenergan 25 mg. is equipotent with Anthisan 150 mg. and Histantin 50 to 100 mg. Because of the variation in length of action of the drugs, after repeated doses have been used for a few days, a daily dose of Phenergan 25 mg. is equipotent with Anthisan 350 mg. and Histantin 100 mg.

4. Individual patients vary considerably in the degree of histamine antagonism given by the same dose of one of the drugs and also in the length of time that the effect of the drug will remain.

5. Fluctuation in the stimuli provoking a disorder such as urticaria may necessitate an alteration in dosage of the drugs to maintain the same response.

6. Amongst dermatological disorders only those with urticarial weal formation are irrefutably improved. In the eczema-dermatitis group it seems likely that a definite effect occurs only when dermal oedema is apparent, as is commonly seen in sensitization dermatitis. It is suggested that there is no specific anti-pruritic action.

### REFERENCES

Bain, W. A. (1949).—*Proc. R. Soc. Med.*, **42**, 615.

Bain, W. A., Broadbent, J. L., and Warin, R. P. (1949).—*Lancet*, **2**, 47.

Bain, W. A., Hellier, F. F., and Warin, R. P. (1948).—*Lancet*, **2**, 964.

Warin, R. P. (1948).—*Brit. Med. Journ.*, **2**, 169.

Warin, R. P. (1950).—*Brit. J. Derm. Syph.*, **62**, 159.

